# Assessment of community knowledge on non-communicable diseases to inform the pilot of WHO PEN-Plus initiatives in selected two districts in Tanzania

**DOI:** 10.1371/journal.pone.0321695

**Published:** 2025-04-15

**Authors:** Gibson B. Kagaruki, Peter Mathias Karoli, Willfredius M. Rutahoile, Pilly Chillo, Reuben Mutagaywa, Aidan Banduka, Edna Siima Majaliwa, Renatus Fabiano Nyarubamba, Esther Mtumbuka, Elizabeth Mallya, Katunzi Mutalemwa, Noemi Bazzanin, Deogratias Soka, Agnes Jonathan, Sarah Urasa, Best Magoma, Lucas J. Kazingo, Heriel Z. Ammi, Emiliana K. Donald, David Raymond Mwenesano, Kajiru Kilonzo, Amani T. Mori, Kaushik Ramaiya, Mayige T. Mary

**Affiliations:** 1 National Institute for Medical Research-Tanzania (NIMR),; 2 Benjamin Mkapa Hospital (BMH),; 3 Second Affiliated Hospital of Chongging Medical University, P.R.China; 4 Muhimbili University of Health and Allied Sciences (MUHAS),; 5 Jakaya Kikwete Cardiac Institute (JKCI),; 6 Muhimbili National Hospital (MNH),; 7 The Tanzania NCD Alliance (TANCDA),; 8 Tanzania Diabetes Association (TDA),; 9 Clinton Health Access Initiative (CHAI),; 10 Doctors with Africa CUAMM,; 11 Tanzania Sickle Cell Disease Foundation (TANSCDF),; 12 Kilimanjaro Christian Medical University College (KCMUCo),; 13 Karatu District Hospital,; 14 Karatu Lutheran Hospital,; 15 Kondoa Town Council,; 16 National Health Insurance Fund (NHIF),; 17 Kilimanjaro Christian Medical Center (KCMC),; 18 University of Bergen,; 19 Dodoma Regional Hospital; Freelance Consultant, Myanmar, MYANMAR

## Abstract

**Background:**

Non-communicable diseases (NCDs) have increased significantly in Tanzania, accounting for 33% of mortality in the country. Having adequate knowledge translated into practice has a significant effect on the health of individuals through adoption of positive behaviours and influencing better health seeking behaviours. For those already affected by NCDs, it promotes secondary and tertiary prevention by helping them effectively cope with the disease. In this study, we aimed to determine the level and determinants of NCDs knowledge in the community to inform the implementation of WHO PEN- Plus initiatives.

**Methods:**

This cross-sectional study was conducted from May to June 2023 involving 528 adults aged 25–64 years from two purposely selected districts and 11, 22 and 528 randomly selected wards, villages, and households respectively. Information on socio-economic, demographic, NCDs knowledge were collected from each participant. Chi-square test and Modified Poisson Regression were applied to assess the association and determinants of NCDs knowledge level.

**Results:**

The median age of study participants was 40.5 years. Less than half 42.6%(n=225) were aware of the term NCD and less than one third were aware of the NCD conditions such as Type 1 Diabetes Mellitus (T1DM) 15.3%(n=70), Sickle Cell Disease (SCD) 25.2%(n=133), Rheumatic fever 28.6%(n=151) and Heart failure 33.1%(n=175). Good level of awareness was reported on Type 2 Diabetes Mellitus (T2DM) 79.5%(n=364). More than three quarters of the respondents had low knowledge on T1DM (90.3%), SCD (84.1%), Rheumatic fever (81.3%), NCDs (80.5%) and Heart failure (76.1%). Furthermore, more than half (56.4%) of respondents had low knowledge for T2DM. Adjusted analysis indicated that, for all NCDs except SCD, low knowledge was significantly associated with the district of residence (Kondoa). Low knowledge of NCDs, T2DM, and SCD was significantly associated with having no education, or having only primary or secondary education. Individuals from the lowest, second, middle, and fourth socio-economic status families were significantly associated with low knowledge of NCDs, while the lowest and fourth socio-economic status levels were associated with low knowledge of T2DM and SCD.

**Conclusion:**

Low knowledge was observed for all NCDs conditions and socio-economic and demographic characteristics were associated with low knowledge. Interventions to increase NCDs knowledge should consider the socio-economic determinants.

## Introduction

The burden of non-communicable diseases (NCDs) is alarming at global, regional and local levels [1]. The surge is responsible for more than two thirds of total global deaths and account for about 40% premature deaths and majority (82%) of premature deaths are from low‐ and middle‐income countries [1,2]. In Tanzania, NCDs accounted for 33% of all deaths in 2016 and they are projected to surpass the burden of deaths by infectious diseases in a few years to come [3]. The prevalence of NCDs including Type 1 Diabetes, Type 2 Diabetes, Rheumatic Heart Diseases (RHD) and its precursors (Sore throat, Rheumatic fever), Heart failure and Sickle Cell Disease (SCD) is increasing exponentially. For example, the number of people living with diabetes in Sub Saharan Africa (SSA) is expected to increase from 16 million in 2017–41 million in 2045; this increase is equivalent to 156% and nearly three quarters of deaths from diabetes occur among individuals aged below 60 years old. Furthermore, about 69% of diabetes cases are undiagnosed and, on many occasions, T2DM patients are diagnosed late with acute complications including kidney failure, amputation, blindness, stroke, impotence, and deaths which further impose cost burden in the health system and at the family level [3–5]. Furthermore, the International Diabetes Federation (IDF) estimates up to 1.2 million children and adolescents have Type 1 diabetes, however, most die undiagnosed, hence the unknown number of cases[6]. Of the data available in Tanzania, the incidence of type 1 diabetes is at 1.8–1.9/100,000 population[6]. This is a very low rate compared to the 52.2/100,000 population in Scandinavian countries[6,7]. Patients with RHD seek medical attention when already in the late stages of their illness, hence presenting with complications (heart failure, infective endocarditis, atrial fibrillation, pulmonary hypertension, stroke etc). In 2010, 48% of patients who underwent open heart surgery at the Jakaya Kikwete Cardiac Institute (the national cardiac hospital) were due to RHD and many of these patients were young (mean age 19.4±12.3 years[8]. The management of these patients at late stages is associated with high-cost implications at family level as well as in the health system leading to poor treatment outcomes including disability and deaths[8]. Reasons proposed for the low incidence rate of T1DM in Tanzania were low knowledge in both the community and health care providers, high mortality due to misdiagnosis by health care providers, low public awareness and myths about diabetes in children together with mismanagement due to lack of knowledge as well as limited capacity of health system in detecting and managing NCDs [9]. Comprehensive knowledge of the disease involves knowing its causes or exacerbating factors, signs and symptoms, prevention measures, available treatments, and consequences, all these are of paramount importance in disease prevention. Such knowledge is crucial to every individual before and or after being-diagnosed with the disease to ensure better outcomes of treatment or intervention for healthy and diseased individuals respectively[10,11]. For the person with the disease, having adequate knowledge of the disease promotes secondary and tertiary prevention by helping an individual to effectively adapt and cope with the disease without stress[10]. Therefore, there is a need for studies that explore the level of NCDs awareness and knowledge, and the factors associated with low knowledge as well as gaps to be addressed. In 2019, WHO AFRO adopted the PEN-Plus strategy, which was an extension of the PEN strategy to address severe NCDs through integrated outpatient clinics at first level referral health facilities. The Ministry of Health (MoH) through the National Institute for Medical Research (NIMR) in Tanzania is piloting initiatives in Kondoa and Karatu districts from Dodoma and Arusha regions respectively. One of the objectives of the pilot is “to assess community levels of knowledge on NCDs at baseline and at the end of project implementation”. However, before commencing community NCDs awareness and knowledge creation campaigns, the program started with conducting baseline evaluation to inform the programmatic activities and to generate baseline indicator values. This study therefore presents information on level of NCDs awareness, knowledge and factors associated with low level of knowledge for NCDs among community members.

## Methods

### Study design and setting

This was a cross-sectional baseline assessment among community members aged 25–64 years, which is the age bracket prioritized by WHO for Non-communicable diseases prevention [12]. The study was carried out in Kondoa district in Dodoma Region and Karatu district in Arusha region between May to June 2023. These two rural districts were purposely selected since the pilot study for the WHO PEN-Plus initiatives is being conducted in these administrative areas in Tanzania. Kondoa districts has a total population of 223,153 while the population of Karatu was 222,637 according to the national census survey of 2022[13]. Karatu district has a total of 66 public and private health facilities (dispensaries=54, health centres=9 and hospital=3) while Kondoa district has 34 dispensaries, 8 health centres, and 1 hospital. The districts were selected for the pilot study due to their higher prevalence of Rheumatic Heart Disease and Sickle Cell Disease, as indicated by the number of cases attending tertiary hospitals. This suggests an increased presence of risk factors and potentially limited knowledge in the community regarding the prevention and management of NCDs. These factors triggered the need for conducting a baseline assessment to determine community level of knowledge on example of NCDs, risk factors, prevention measures, sign and symptoms, treatment and complications of selected NCDs including NCDs in general, Type 1 Diabetes, Type 2 Diabetes, Rheumatic fever, Heart failure and Sickle Cell Diseases.

### Sample size calculation

Since the aim of this baseline cross sectional study design was to generate baseline information to inform interventional initiatives for the WHO PEN-Plus; a recommended formula for intervention was used to calculate a sample with power to determine the desired effect size of the project [14–16]. The following statistical parameters were considered in calculating the sample size: 50%, a conservative value as the proposed baseline value of indicator (P1) the % of community members with adequate NCDs knowledge level, we also assumed an effect size of 12 percent for a P_2_ = 62 percent and power of 80 percent. The average of P_1_ and P_2_, i.e., P = 56 percent. Other parameters for sample size calculation include the Design Effect (DE) = 2 and a 20 percent attrition/non-response rate was considered, based on the above assumptions and considering proportion allocation then our actual sample size, n=528. The formula for calculating sample size for cross sectional study also resulted in the same sample size. This sample size was distributed proportionally considering the population size aged 25–64 years in each target district. In our case, in 2021 a total of 168,808 and 142,105 people aged 25–64 years were estimated from Kondoa and Karatu districts respectively.

### Sampling procedures

A multistage cluster sampling that involved both purposive and probability sampling was applied in selecting the study sites and respondents. Initially, two districts namely Kondoa and Karatu, were purposefully selected as the only administrative areas where the WHO PEN-Plus initiatives are now being implemented [17]. Within each district, a list of wards was obtained and six (6) wards in Kondoa and five (5) wards in Karatu were randomly selected. From each ward, two villages/streets were also selected randomly after obtaining a list of available units from the ward executive officer of a respective ward. In total, 22 villages/mtaas (10 from Karatu and 12 from Kondoa) were selected for this study. Selection of villages/mtaa was followed with selection of households. In a selected village/mtaa, the team applied systematic sampling to select the households from that sampling frame. The first stage involved determining the sampling interval and this was obtained by dividing the total number of households with at least one adult members aged 25–64 years in that street/village by the required number of sample size to be selected in that village which was 24. That is the total number of households divided by 24. For instance, if a village had 120 households with at least one individual aged 25–64 years, subsequently, the figure was divided by 24 the required number of households per village/street then the sampling interval was 5. However, the team found that the village/mtaas officer did not have an up-to-date list of households, due to that setback, the village/mtaas officer was requested to estimate the number of households based on his/her best knowledge about the area. After establishing the sampling interval, the team determined the starting household by going to the central location within the communities, e.g., village office, market, church, heath facility or junction between two roads gained by “spin bottle approach” to decide the direction. The nearest house was then selected as the first house, then the next houses were subsequently selected following the computed sampling interval based on the estimated number of households in the villages/mtaas (which was determined with the assistance of the village/mtaas office leaders). In each sampled household, the data collector was required to list all eligible household members and select one individual randomly using a randomization algorithm that was inbuilt in the electronic data collection software. In case a selected household had no eligible household member it was replaced with the nearby ones.

### Study variables

This study had the following independent variables: District (Kondoa & Karatu), Gender (Male & Female), Age group (25–34, 35–44, 45–54 & 55–64) years, Education (None/primary, Secondary & College), Marital status (Married & Not married), occupation (Employed, Self-employed, Unemployed and Others) and SES (Lowest, Second, Middle, Fourth and Highest)[18,19]. A total of 11 items were used to assess the household socio-economic status (SES). These items included living in a corrugated house with iron sheets or concrete, having tap water as the main source of drinking water, and being connected to the national grid for electricity supply. Other items assessed included owning the following assets: television, radio, iron, mobile phone, and having any household member with a bank account. Additionally, the assessment considered using a standard type of toilet, living in a house with exterior walls made of stones or blocks, and having electricity as the main source of lighting, connected to the national grid. These items were adopted from the TDHS surveys[18,19]. A weight of “1” was given if the answer was “yes” and “0” if otherwise. Later, the principal component analysis statistical technique was applied to cluster the composite indicator (i.e., SES) into five quintiles: Lowest, Second, Middle, Fourth, and Highest[18,20]. The outcome variables of interest were: Having low, moderate and high knowledge on NCDs in general, Type 1 Diabetes, Type 2 Diabetes, Rheumatic fever, Heart failure and Sickle Cell Disease. Each disease/domain had sub-domains, and each sub-domain had items assessed. For instance, six subdomains were assessed under Type 1 Diabetes. The following are subdomains assessed, and the respective number of items evaluated in bracket: Causes of type 1 diabetes (2), Type 1 diabetes risk factors (14), Symptoms of type 1 diabetes (8), Complications of type 1 diabetes (10), Treatment of type 1 diabetes (10) and Prevention measures of complication for type 1 diabetes (6). A respondent was considered to have adequate knowledge on a subdomain if was able to mention at least 1 out of 2 items for Causes of type 1 diabetes, at least 2 out of 14 items for Type 1 diabetes risk factors, at least 2 out of 8 for Symptoms of type 1 diabetes, at least 1 out of 10 for Complication of type 1 diabetes, at least 1 out of 10 for Treatment of type 1 diabetes and at least 1 out of 6 for Prevention measures of complications for type 1 diabetes. Those who had adequate knowledge on a respective subdomain were given a score/weight of “1” and “0” if otherwise, these scores/weights were aggregated to get an individual overall domain (Type 1 Diabetes) score which ranged from 0–6 scores. Later, an overall score of each respondent was categorized into low, moderate and high knowledge if an individual’s scores were 0–2, 3–4 and 5–6 respectively [21]. Details for other diseases domain, sub-domain, items, scoring and categorization see Appendix A.

### Training of field team

The study’s principal investigator (PI) and co-principal investigators (Co-PIs) prepared the training materials for the research assistants (RAs) and field supervisors. Typically, this includes a slide deck summarizing the field manual and scenarios for role-playing. After receiving ethical approval, the PI and Co-PIs conducted a three-day, theory- and practice-based training (including role-playing) and study kick-off session for the RAs. The training involved 12 field team members (10 data collectors and two supervisors) and was led by the PI and Co-PIs. The training covered the following topics: an overview of the project and survey methodology, data collection strategy, participant recruitment and sampling approach, data collection tools, ethical considerations, quality assurance methods, data security plan, and team roles, management, and coordination.

### Pilot testing

As a standard practice, the PI and Co-PIs pilot test the data collection tool, respondent recruitment strategy, and field logistics process for each study with target respondents. Additionally, the pilot test allows the study team to generate data for a quick initial analysis of randomly selected study indicators or questions, helping to validate the data mapping exercise as well as refining the data collection tool. This serves as another effort to ensure that the programmed questionnaire produces the intended data. Finally, the pilot test provides an opportunity for the trained RAs to practice what they have learned. The pilot test was conducted in a mtaa/street called Sinza Moli in the Kinondoni district of Dar es Salaam city to simulate the protocol requirements and field logistics plan.

### Refinement of data collection tool & field manual

Upon completion of the pilot test, the study team debriefed on the research implementation process, including the respondents’ comprehension of the questions, the flow of the questions, and the identification of any questions that may need refinement in the data collection tools. The study statistician ran a quick analysis of the data collected during the pilot test to ensure that the questionnaires aligned with the indicators the study intended to collect.

### Data collection, management, cleaning and analysis

The data collection was done electronically using Open Data Kit (ODK) software which was programmed and installed in the tablets. Data quality was mainstreamed throughout the entire period of the study, being emulated to ensure that the research findings are of high quality and adhere to sound ethical principles. Data quality check was implemented during field survey and before actual data analysis. Besides, consistency checks were built into the data captured using Kobo toolbox to ensure that no missing information or implausible values were accepted. For instance, the data collection system included controls for variables range, skips, duplicated individuals, and intra- and inter-module consistency checks to reduce or eliminate errors usually introduced at the data capture stage. To ensure data quality, the Data Manager always made sure that daily reports on the data collected matched the ones in the server. Access to the data and the database was granted to only authorized research members. After data collection we cleaned the data to detect duplicates, inconsistent responses, outliers as well as non-systematic missing data. Prior to downloading the data from the cloud server, all tablets were checked to ensure that all completed questionnaires had been uploaded to the server. The complete dataset was downloaded in a.csv (Comma Separated Value) format. Data downloaded from the server were then exported from Excel to Stata version 17 (STATA Corp Inc., TX, USA) for analysis. Data analysis started with running descriptive statistics to describe the characteristics of the data set. This included running the univariate analysis on frequency and percentage response distributions and measures of central tendency and dispersion. Later, we conducted inferential analysis using appropriate methods (e.g., Chi-squares and Modified Poisson logistic regression). Modified Poisson Regression Model was opted since the proportion of community member with low knowledge on NCDs in general, Type 1 Diabetes, Type 2 Diabetes, Rheumatic fever, Heart failure and Sickle cell disease was greater than 10% [22,23]. An explanatory variable was included in the adjusted analysis at p-value<0.2 but considered significant if p-value<0.05 in the adjusted analysis and this study had reported both Unadjusted and Adjusted Prevalence Ratios.

### Ethical considerations

This study followed a set of ethical principles in accordance with the revised 1964 Declaration of Helsinki, including ethical approval and informed consent [24]. Ethical approval to conduct this study was obtained from the National Institute for Medical Research NIMR, reference number NIMR/HQ/R.8a/Vol.IX/4184. Permission to conduct the study was obtained from President’s Office, Regional Administration and Local Government Tanzania (PORALG), District Medical Officer of Kondoa and Karatu districts as well as from wards and village/street executive officers. All the study participants provided written informed consent to participate in the study, ensured the confidentiality of their responses and the interview with each respondent was done in the areas with privacy.

## Results

### Demographic information

Table 1 displays the demographic and socio-economic characteristics of the study participants. The majority of respondents were female (289, 54.7%), married (364, 68.9%), and self-employed (368, 69.7%). The median age of respondents in both districts was 39 years, with the majority falling between the ages of 25–44, representing over 64% of the sample. Most participants had primary education or no education (77%), with very few having a college education (5.3%). The difference in primary or no education was more pronounced in Kondoa. The majority of respondents were in the middle or fourth socio-economic strata (40.2%), while 36.7% and 20.1% were in the highest and lowest strata, respectively. Generally, Kondoa had fewer educated participants and a less wealthy population.

**Table 1:** Socio-economic and demographic characteristics of the respondents by district (n=528).

Variable	Category	Kondoa, n(%)	Karatu, n(%)	Total, n(%)
Gender	Male	135(46.9)	104(43.3)	239(45.3)
	Female	153(53.1)	136(56.7)	289(54.7)
Age	Median (IQR)	40.5(33,52.5)	36(29.5,47)	39(31,50)
Age group	25-34	82(28.5)	109(45.4)	191(36.2)
	35-44	80(27.8)	65(27.1)	145(27.5)
	45-54	63(21.9)	34(14.2)	97(18.4)
	55-64	63(21.9)	32(13.3)	95(18.0)
Education	None/primary	253(87.9)	155(64.6)	408(77.3)
	Secondary	28(9.7)	64(26.7)	92(17.4)
	College	7(2.4)	21(8.7)	28(5.3)
Marital status	Single	17(5.9)	58(24.2)	75(14.2)
	Married	212(73.6)	152(63.3)	364(68.9)
	Separated	25(8.7)	17(7.1)	42(8.0)
	Divorced	2(0.7)	2(0.8)	4(0.8)
	Widowed	20(6.9)	10(4.2)	30(5.7)
	Cohabiting	12(4.2)	1(0.4)	13(2.5)
Occupation	Government employee	5(1.7)	10(4.2)	15(2.8)
	Non-government employee	1(0.4)	16(6.7)	17(3.2)
	Self-employed	196(68.1)	172(71.7)	368(69.7)
	Retired	2(0.7)	1(0.4)	3(0.6)
	Unemployed	65(22.6)	21(8.8)	86(16.3)
	Student/pupil	1(0.4)	1(0.4)	2(0.4)
	Others	1(0.4)	19(7.9)	37(7.0)
SES	Lowest	94(32.6)	12(5.0)	106(20.1)
	Second	60(20.8)	46(19.2)	106(20.1)
	Middle	64(22.2)	41(17.1)	105(19.9)
	Fourth	53(18.4)	53(22.1)	106(20.1)
	Highest	17(5.9)	88(36.7)	105(19.9)
	Total	288(100)	240(100)	528(100)

### NCDs, type 1 diabetes, type 2 diabetes, rheumatic fever, heart failure and sickle cell disease awareness

Less than half 42.6%(n=225) were aware of the term NCD and less than one third were aware of the NCD conditions such as type 1 diabetes 15.3%(n=70), Sickle Cell Disease 25.2%(n=133), Rheumatic fever 28.6%(n=151) and Heart failure 33.1%(n=175). However, a good level of awareness was documented on T2DM 79.5%(n=364).

### Level of knowledge on NCDs, type 1 diabetes, type 2 diabetes, rheumatic fever, heart failure and sickle cell disease

**Fig 1:**
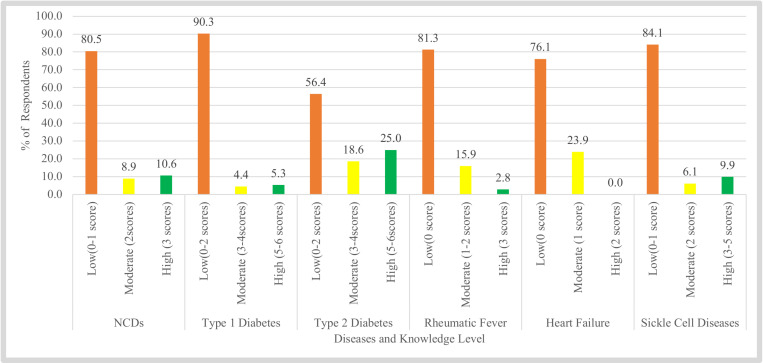
Proportion of respondents with low, moderate and high knowledge on NCDs (n=528).

More than three quarters of the respondents had low knowledge on Type 1 Diabetes (90.3%), Sickle Cell (84.1%), Rheumatic fever (81.3%), NCDs (80.5%) and Heart failure (76.1%). Furthermore, more than half (56.4%) of respondents had low knowledge for type 2 diabetes. None had high knowledge on heart failure and less than 10% had high knowledge on NCDs, Type 1 Diabetes, Rheumatic fever and Sickle Cell. A quarter of respondents had high knowledge on Type 2 diabetes (Fig 1).

### Association between knowledge and socio-demographic factors

Bivariate analysis using Pearson Chi-Square and Chi-Square for trend to assess the association between NCD related knowledge and socio-economic and demographic factors showed that participants from Kondoa, those with no formal or primary education, and those from low SES households were more likely to have low NCD knowledge compared to the comparative groups. Low knowledge of type 1 diabetes, type 2 diabetes, rheumatic fever, heart failure, and sickle cell disease was significantly observed among individuals from Kondoa, those with no formal or primary education, and those from households with the lowest SES compared to the respective counterpart groups. Regarding occupation, unemployed individuals were more likely to have low knowledge of NCDs, T2DM, rheumatic fever, and sickle cell disease compared to employed individuals (Table 2).

**Table 2:** Association between low knowledge on knowledge on NCDs and socio-economic and demographic factors: bivariate analysis.

Variable	N	NCDs, n(%)	Type 1 Diabetes, n(%)	Type 2 Diabetes, n(%)	Rheumatic fever, n(%)	Heart failure, n(%)	Sickle Cell Disease n(%)
District							
Kondoa	288	273(94.8)^**^	278(96.5)^**^	214(74.3)^**^	280(97.2)^**^	210(72.9)^**^	259(89.9)^**^
Karatu	240	152(63.3)	199(82.9)	84(35.0)	149(62.1)	118(49.2)	185(77.1)
Gender							
Male	239	196(82.0)	219(91.6)	129(54.0)	198(82.9)	151(63.2)	195(81.6)
Female	289	229(79.2)	258(89.3)	169(58.54)	231(79.9)	177(61.3)	249(86.2)
Age group							
25-34	191	150(78.5)	173(90.6)	104(54.5)	153(80.1)	121(63.4)	154(80.6)
35-44	145	110(75.9)	130(89.7)	82(56.6)	120(82.8)	87(60.0)	123(84.8)
45-54	97	83(85.6)	85(87.6)	56(57.7)	79(81.4)	61(62.9)	84(86.6)
55-64	95	82(86.3)	89(93.7)	56(59.0)	77(81.1)	59(62.1)	83(87.4)
Education							
None/primary	408	359(88.0)^**^	382(93.6)^**^	259(63.5)^**^	349(85.5)^**^	267(65.4)^**^	369(90.4)^**^
Secondary	92	56(60.9)	75(81.5)	37(40.2)	65(70.7)	49(53.3)	63(68.5)
College	28	10(35.7)	20(71.4)	2(7.1)	15(53.6)	12(42.9)	12(42.9)
Marital status							
Married	88	67(76.1)	76(86.4)	43(48.9)	74(84.1)	52(59.1)	72(81.8)
Not married	440	358(81.4)	401(91.1)	255(58.0)	355(80.7)	276(62.7)	372(84.6)
SES							
Lowest	106	103(97.2)^**^	103(97.2)^**^	88(83.0)^**^	103(97.2)^**^	77(72.6)^**^	98(92.5)^**^
Second	106	97(91.5)	98(92.5)	65(61.3)	94(88.7)	63(59.4)	96(90.6)
Middle	105	90(85.7)	99(94.3)	64(61.0)	87(82.9)	78(74.3)	97(92.4)^**^
Fourth	106	84(79.3)	93(87.7)	47(44.3)	76(71.7)	57(53.8)	83(78.3)
Highest	105	51(48.6)	84(80.0)	34(32.4)	69(65.7)	53(50.5)	70(66.7)
Occupation							
Employed	32	16(50.0)	26(81.3)	7(21.9)	16(50.0)	14(43.8)	19(59.4)
Self-employed	368	298(81.0)	336(91.3)	212(57.6)	305(82.9)	240(65.2)^	319(86.7)
Unemployed	86	78(90.7)^**^	79(91.9)	57(66.3)^**^	78(90.7)^**^	52(60.5)	76(88.4)^**^
Others	42	33(78.6)	36(85.7)	22(52.4)	30(71.4)	22(52.4)	30(71.4)

***P-value:***

^ ***<0.05and***

** ***<0.001***

In multivariable analysis, the results indicated that individuals from Kondoa district were significantly more likely to have low knowledge of NCDs (APR=1.3; 95%CI 1.2–1.4), type 1 diabetes (T1DM) (APR=1.1; 95%CI 1.01–1.2), type 2 diabetes (T2DM) (APR=1.8; 95%CI 1.5–2.2), rheumatic fever (APR=1.5; 95%CI 1.3–1.7), and heart failure (APR=1.4; 95%CI 1.2–1.7) compared to those from Karatu district. Individuals with no formal education, or only primary or secondary education, were more likely to have low knowledge of NCDs (APR=2.0; 95%CI 1.2–3.0) and (APR=1.67; 95%CI 1.03–3.33), low knowledge of type 2 diabetes (APR=6.3; 95%CI 1.7–23.5) and (APR=5.34; 95%CI 1.5–19.7), and low knowledge of sickle cell disease (APR=1.9; 95%CI 1.3–3.0) and (APR=1.6; 95%CI 1.0–2.5), as compared with those with a college education. Additionally, individuals in the lowest, second, middle, and fourth SES groups were more likely to have low knowledge of NCDs (APR=1.5; 95%CI 1.2–1.8), (APR=1.5; 95%CI 1.2–1.8), (APR=1.4; 95%CI 1.1–1.8), and (APR=1.4; 95%CI 1.1–1.8) compared to those from households with highest SES, respectively. Significant low knowledge of T2DM was observed among participants in the lowest SES (APR=1.4; 95%CI 1.02–1.9) compared to those in the highest SES (Tables 3 and 4).

**Table 3:** Multivariable analysis to determine the factors associated with low NCDs, type 1 diabetes, type 2 diabetes, rheumatic fever, heart failure and sickle cell knowledge.

Variable	NCDs		Type 1 Diabetes		Type 2 Diabetes	
	UPR,95%CI	APR,95%CI	UPR,95%CI	APR,95%CI	UPR,95%CI	APR,95%CI
District						
Kondoa	1.5(1.4-1.7)^**^	1.3(1.2-1.4)^**^	1.2(1.1-1.2)^**^	1.1(1.04-1.2)^*^	2.1(1.8-2.6)^**^	1.8(1.5-2.2)^**^
Karatu	1	1	1	1	1	1
Gender						
Male	1.0(1.0-1.1)		1.0(1.0-1.1)		0.9(0.8-1.1)	
Female	1		1		1	
Age group						
25-34	1		1		1	
35-44	1.0(.9-1.1)		1.0(0.9-1.1)		1.0(0.9-1.3)	
45-54	1.1(1.0-1.2)		1.0(0.9-1.1)		1.1(0.9-1.3)	
55-64	1.1(1.0-1.2)		1.0(0.9-1.1)		1.1(0.9-1.3)	
Education						
None/primary	2.5(1.5-4.1)^**^	2.0(1.2-3.3)^*^	1.3(1.03-1.7)^	1.2(1.0-1.5)	8.9(2.3-33.9)^*^	6.2(1.6-23.5)^*^
Secondary	1.7(1.0-2.9)^	1.7(1.03-3.3)^	1.1(0.9-1.5)	1.1(0.9-1.4)	5.6(1.4-21.9)^	5.3(1.4-19.7)^
College	1	1	1	1	1	1
Marital status						
Married	1		1	1	1	1
Not married	1.1(0.9-1.2)		1.1(1.0-1.2)		1.2(0.9-1.5)+	0.9(0.7-1.1)
SES						
Lowest	2.0(1.6-2.4)^**^	1.5(1.2-1.8)^**^	1.2(1.1-1.3)^**^	1.1(0.9-1.2)	2.6(1.9-3.4)^*^	1.4(1.02-1.9)^
Second	1.9(1.5-2.3)^**^	1.5(1.2-1.9)^**^	1.2(1.0-1.3)^	1.0(0.9-1.2)	1.9(1.4-2.6)^*^	1.2(0.9-1.7)
Middle	1.8(1.4-2.2)^**^	1.4(1.1-1.8)^*^	1.2(1.1-1.3)^*^	1.1(1.0-1.2)	1.9(1.4-2.6)^*^	1.2(0.9-1.7)
Fourth	1.6(1.3-2.0)^**^	1.4(1.1-1.8)^*^	1.1(1.0-1.2)+	1.0(0.9-1.2)	1.4(1.0-1.9)+	1.0(0.7-1.4)
Highest	1	1	1	1	1	1
Occupation						
Employed	1	1	1	1	1	1
Self-employed	1.6(1.2-2.3)^**^	0.9(0.6-1.3)	1.1(0.9-1.3)		2.6(1.4-5.1)^**^	1.0(0.5-2.0)
Unemployed	1.8(1.3-2.6)^**^	0.9(0.6-1.4)	1.1(0.9-1.4)		3.0(1.5-5.9)^**^	1.0(0.5-2.0)
Others (Students, retired etc)	1.6(1.1-2.3)^	0.9(0.6-1.4)	1.1(0.9-1.3)		2.4(1.2-4.9)^	1.0(0.5-2.1)

***P-value:***

^ ***<0.05,***

* ***0.01, and***

** ***<0.001, Unadjusted Prevalence Ratio (UPR), Adjusted Prevalence Ratio (APR), Confidence Interval (CI)***

**Table 4:** Factors associated with low NCDs, type 1 diabetes, type 2 diabetes, rheumatic fever, heart failure and sickle cell knowledge.

Variable	Rheumatic fever		Heart failure		Sickle Cell	
	UPR,95%CI	APR,95%CI	UPR,95%CI	APR,95%CI	UPR,95%CI	APR,95%CI
District						
Kondoa	1.6(1.4-1.7)^**^	1.5(1.3-1.7)^**^	1.4(1.3-1.7)^**^	1.4(1.2-1.7)^**^	1.2(1.1-1.3)^**^	1.1(1.0-1.1)
Karatu	1	1	1	1	1	1
Gender						
Male	1.0(1.0-1.1)		1.0(0.9-1.2)		0.9(0.9-1.0)+	1.0(0.9-1.0)
Female	1		1	1	1	1
Age group						
25-34	1		1		1	1
35-44	1.0(0.9-1.1)		0.9(0.8-1.1)		1.1(1.0-1.2)	1.0(0.9-1.1)
45-54	1.0(0.9-1.1)		1.0(0.8-1.2)		1.1(1.0-1.2)+	1.0(0.9-1.0)
55-64	1.0(0.9-1.1)		1.0(0.8-1.2)		1.1(1.0-1.2)+	1.0(0.9-1.1)
Education						
None/primary	1.6(1.1-2.3)^*^	1.2(0.8-1.7)	1.5(1.0-2.4)+	1.2(0.7-2.0)	2.1(1.4-3.2)^*^	1.9(1.3-3.0)^*^
Secondary	1.3(0.9-1.9)+	1.2(0.8-1.7)	1.2(0.8-2.0)	1.1(0.7-1.8)	1.6(1.0-2.5)^	1.6(1.01-2.5)^
College	1	1	1	1	1	1
Marital status						
Married	1		1		1	
Not married	1.0(0.9-1.1)		1.1(0.9-1.3)		1.0(0.9-1.2)	
SES						
Lowest	1.5(1.3-1.7)^**^	1.01(0.9-1.2)	1.4(1.2-1.8)^*^	1.1(0.8-1.4)	1.4(1.2-1.6)^**^	1.1(1.0-1.3)
Second	1.4(1.2-1.6)^**^	1.04(0.9-1.2)	1.2(0.9-1.5)+	0.9(0.7-1.3)	1.4(1.2-1.6)^**^	1.1(1.0-1.3)
Middle	1.3(1.1-1.5)^**^	1.0(0.8-1.2)	1.5(1.2-1.8)^*^	1.2(0.9-1.5)	1.4(1.2-1.6)^**^	1.2(1.02-1.4)^
Fourth	1.1(0.9-1.3)	0.9(0.7-1.1)	1.1(0.8-1.4)	0.9(0.7-1.2)	1.2(1.0-1.4)+	1.1(0.9-1.3)
Highest	1	1	1	1	1	1
Occupation						
Employed	1	1	1	1	1	1
Self-employed	1.7(1.2-2.4)^**^	1.3(0.9-1.9)	1.5(1.0-2.2)^	1.2(0.7-1.8)	1.5(1.1-2.0)^	1.0(0.7-1.3)
Unemployed	1.8(1.3-2.6)^**^	1.3(0.9-1.9)	1.4(0.9-2.1)	1.0(0.6-1.6)	1.5(1.1-2.0)^**^	1.0(0.7-1.4)
Others (Students, retired etc)	1.4(1.0-2.1)+	1.1(0.8-1.7)	1.2(0.7-2.0)	1.0(0.6-1.6)	1.2(0.9-1.9)	0.8(0.6-1.2)

*P-value:*

^ *<0.05,*

* *0.01, and*

** *<0.001, Unadjusted Prevalence Ratio (UPR), Adjusted Prevalence Ratio (APR), Confidence Interval (CI)*

## Discussion

Awareness is the first step towards acquiring knowledge, but knowledge goes beyond mere awareness, involving a deeper and more thorough understanding of a subject. It should be noted that, high level of awareness does not necessarily imply a high level of knowledge [25,26]. The current study aimed to assess awareness, knowledge, and factors contributing to low knowledge of non-communicable diseases (NCDs) among community members. It focused on key aspects of NCDs, including risk factors, prevention, signs, symptoms, treatment, and complications. Findings revealed generally low awareness and low knowledge levels across various NCDs. Disparities were noted between districts, education levels, and socio-economic statuses. Diabetes Mellitus emerged as the most known NCD compared to other NCDs, but emphasis is needed on other conditions, especially among the poor and marginalized populations, including children. Tanzania, with a high prevalence of Sickle Cell Disease (SCD) and other NCDs, requires increased community awareness for preventive services and early screening, particularly among vulnerable groups. Low NCD knowledge was associated with demographic factors such as education, location, and socio-economic status.

The current observed findings may be explained by a historical background that has persisted for a long time in developing countries [27]. Developing countries, including Tanzania, have historically grappled with communicable diseases such as HIV, Malaria, TB, prompting significant investment in prevention and treatment measures. However, there has been a notable shift in the disease burden towards non-communicable diseases (NCDs) due to exponential increase of both environmental and biological risk factors [28–31]. In Sub-Saharan Africa, NCDs now surpass communicable diseases in terms of causing deaths and overall disease burden[28]. Despite this, communities lack adequate knowledge and skills to address NCDs effectively, resulting in the majority being diagnosed late[21,32]. Late diagnosis often leads to severe complications like blindness, amputation, stroke, and kidney failure, imposing substantial cost burdens on families and healthcare systems [3]. The findings underscore the importance of allocating resources for NCD prevention through various awareness channels, such as television, social media, and community gatherings.

Moreover, there’s a call for integrating NCDs with well-funded programs like HIV/TB to optimize resource allocation and address the underfunding of NCD initiatives [33,34]. It is also high time to design and implement intervention programs such as WHO PEN-Plus in rural settings where the capacity and capability of detecting and managing NCDs have been reported to be limited despite an increase in the burden in these areas. Such interventions are highly needed in Tanzania, where NCDs are responsible for 33% of deaths[3]. These intervention programs have been proved to be feasible in Tanzania. For example, in this study, we observed that the level of knowledge for diabetes was relatively higher compared to other NCD conditions, which might be due to national diabetes programs funded by the International Diabetes Federation (IDF) and implemented in Tanzania since 2005, covering all zonal, regional, and district hospitals and some health centres[35]. This program was implemented collaboratively with the Ministry of Health (MOH) and the Tanzania Diabetes Association (TDA) [35]. It is, therefore, hypothesized that programs such as National Diabetes Programs, PEN Plus, etc., coupled with the integration of NCDs and HIV/TB, may contribute to addressing the current observed gaps of awareness and knowledge in the community. The findings from this study are also useful in informing programmatic activities for the WHO PEN Plus initiatives as well as other possible upcoming National NCDs programs. Lastly, this study showed that people with low education, especially those from Kondoa, where most of the respondents had low education, and those coming from poor families, were more likely to have low NCDs knowledge. Similar findings were documented in Dar es Salaam and other areas[36,21 ]. There is a significant need for educational initiatives and campaigns to raise awareness about NCDs, their risk factors, and preventive measures within the community[37]. Another study also conducted in Mwanza region also showed that a big proportion of the study participants had low knowledge on T2DM and very few were aware of the cause of the disease [38].

Furthermore, one of the limitations of this study was the limited number of community-based studies assessing awareness and knowledge levels making it difficult to compare results with this study. Most of the locally available literature/studies are based on specific diseases, such as Type 1 Diabetes, Rheumatic Fever, Heart Failure, and Sickle Cell, and focused on healthcare workers and/or patients. We therefore recommend conducting more community studies on this subject to expand the body of literature and better monitor the effectiveness of ongoing NCD awareness and knowledge creation interventions.

## Conclusion

Low knowledge was observed for all NCDs conditions and socio-economic and demographic characteristics were associated with low knowledge. Interventions to increase NCDs knowledge should consider the socio-economic determinants. It is also recommended that NCDs programs’ awareness and knowledge creation programs should consider channels that will reach people with low socio-economic and education levels. Furthermore, awareness and knowledge are key to preventing NCDs, but these diseases programs have long been underfunded. Integrating NCDs awareness activities with well-funded disease programs like HIV, TB, and Malaria could help increase awareness and knowledge, ultimately contributing to the prevention of NCDs.

## Appendix A: Community knowledge on NCDs, type 1 diabetes, type 2 diabetes, rheumatic fever, heart failure and sickle cell disease

**Table:** 

Domain	Sub-domain	# of items assessed	Score per item	Adequate knowledge	Score for adequate knowledge
NCDs	Example of NCDs	9	1	≥2	1
	NCDs risk factors	10	1	≥2	1
	NCDs prevention measures	6	1	≥2	1
	*Overall NCDs knowledge score*	*Total scores=3, Low<2, Moderate=2 & High=3*			
Type 1 Diabetes	Causes of type 1 diabetes	2	1	≥1	1
	Type 1 diabetes risk factors	14	1	≥2	1
	Symptoms of type 1 diabetes	8	1	≥2	1
	Complication of type 1 diabetes	10	1	≥1	1
	Treatment of type 1 diabetes	10	1	≥1	1
	Prevention measures of complication for type 1 diabetes	6	1	≥1	1
	*Overall T1DM knowledge score*	*Total scores=6, Low<3, Moderate=3–4 & High=5–6*			
Type 2 Diabetes	Know at least 1 cause of type 2 diabetes	2	1	≥1	1
	Type 2 diabetes risk factors	10	1	≥2	1
	Symptoms of type 2 diabetes	9	1	≥2	1
	Complications of type 2 diabetes	10	1	≥1	1
	Treatment of type 2 diabetes	12	1	≥1	1
	Prevention measures of complication for type 2 diabetes	5	1	≥1	1
	*Overall T2DM knowledge score*	*Total scores=6, Low<3, Moderate=3–4 & High=5–6*			
Rheumatic fever	Symptoms of Rheumatic fever	5	1	≥1	1
	Risk factors of Rheumatic fever	6	1	≥1	1
	Prevention measures of Rheumatic fever	7	1	≥1	1
	*Overall Rheumatic fever knowledge score*	*Total scores=3, Low=0, Moderate=1–2 & High=3*			
Heart failure	Signs of heart failure	4	1	≥1	1
	Treatment of heart failure	3	1	≥1	1
	*Overall, Heart failure knowledge score*	*Total scores=2, Low=0, Moderate=1 & High=2*			
Sickle Cell Disease	Cause of sickle cell disease	2	1	≥1	1
	Symptoms of sickle cell disease	4	1	≥2	1
	Complication of sickle cell disease	8	1	≥2	1
	Treatment of sickle cell disease	3	1	≥1	1
	Prevention measures of sickle cell disease	2	1	≥1	1
	*Overall, Sickle Cell Disease knowledge score*	*Total scores=5, Low<2, Moderate=2 & High=3–5*			
